# Comparison of C-reactive protein and procalcitonin as predictors of postoperative infectious complications after elective colorectal surgery

**DOI:** 10.3325/cmj.2012.53.612

**Published:** 2012-12

**Authors:** Dagmar Oberhofer, Josip Juras, Ana Marija Pavičić, Iva Rančić Žurić, Vlatko Rumenjak

**Affiliations:** 1 Department of Anesthesiology and Intensive Care, University Hospital Sveti Duh, Zagreb, Croatia; 2 Department of Obstetrics and Gynecology, Clinical Hospital Centre Zagreb, Zagreb, Croatia; 3 Department of Medical Laboratory Diagnosis, University Hospital Sveti Duh, Zagreb, Croatia

## Abstract

**Aim:**

To assess diagnostic value of perioperative procalcitonin (PCT) levels compared to C-reactive protein (CRP) levels in early detection of infectious complications following colorectal surgery.

**Methods:**

This prospective observational study included 79 patients undergoing elective colorectal surgery. White blood cell count, CRP, and PCT were measured preoperatively and on postoperative days (POD) 1, 2, 3, 5, and patients were followed for postoperative complications. Diagnostic accuracy of CRP and PCT values on each day was analyzed by the receiver operating characteristics (ROC) curve, with infectious complications as an outcome measure. ROC curves with the largest area under the curve for each inflammatory marker were compared in order to define the marker with higher diagnostic accuracy.

**Results:**

Twenty nine patients (36.7%) developed infectious complications. CRP and PCT concentrations increased in the early postoperative period, with a significant difference between patients with and without complications at all measured postoperative times. ROC curve analysis showed that CRP concentrations on POD 3 and PCT concentrations on POD 2 had similar predictive values for the development of infectious complications (area under the curve, 0.746 and 0.750, respectively) with the best cut-off values of 99.0 mg/L for CRP and 1.34 µg/L for PCT. Diagnostic accuracy of CRP and PCT was highest on POD 5, however the cut-off values were not considered clinically useful.

**Conclusion:**

Serial postoperative PCT measurements do not offer an advantage over CRP measurements for prediction of infectious complications following colorectal surgery.

Colorectal surgery leads to high rates of postoperative complications, varying between 28% and 38% (1-3). Early diagnosis and prompt treatment of complications is crucial for a favorable outcome. However, surgical trauma induces systemic inflammatory response syndrome (SIRS), which can hinder the diagnosis of postoperative infections (4). Therefore, it would be useful to find a biochemical marker that could accurately differentiate between infectious and non-infectious SIRS.

C-reactive protein (CRP), the first of the acute phase proteins to be described, was discovered in 1930 and named for its capacity to precipitate a non-protein somatic fraction (Fraction C) of *Streptococcus pneumoniae*. It is a sensitive systemic marker of inflammation and tissue damage, but is not specific for infection (5,6). Surgical trauma induces a significant increase in CRP levels, which can reduce its predictive value for the diagnosis of infection in the early postoperative period (7-9). Despite this, an interest in CRP as an infection monitoring tool in the perioperative setting has increased since it was reported that in values higher than 140 mg/L on the postopertaive day (POD) 3-4 it well predicted infectious complications after colorectal surgery (10). Since then, several studies have found it to be a useful predictor of septic complications following colorectal and esophagogastric resections (11-15).

Procalcitonin (PCT), the prohormone of calcitonin, was first described as a biochemical marker of infection in 1993 (16). Bacterial endotoxins are potent stimuli for PCT synthesis, which exhibits faster kinetics than CRP. PCT is released into the circulation 3-4 hours after an injection of endotoxin, reaching peak levels after 8-24 hours, while CRP peaks at 36-50 hours after stimulus (5,17). This would make PCT more suitable as an infection monitoring tool in the perioperative setting (18,19). A meta analysis of 33 studies, which included adults in intensive care units or after surgery and trauma, showed that PCT was a good diagnostic marker of sepsis, with greater diagnostic accuracy than CRP (20). Recent studies in surgical patients have also shown that after orthopedic, cardiac, and thoracic surgery PCT was better for detecting postoperative infections than CRP (21-23).

To the best of our knowledge, no studies have compared the diagnostic accuracy of CRP and PCT for early detection of postoperative complications in patients undergoing colorectal surgery. The aim of this study was to assess the predictive value of serial postoperative determinations of CRP, PCT, and white blood cell (WBC) count for infectious complications after elective colorectal surgery and to compare the diagnostic accuracy of CRP and PCT.

## Patients and methods

Consecutive patients who underwent elective colorectal surgery and were admitted postoperatively to the surgical intensive care unit (ICU) of a single university hospital between January and December 2009 were included in a prospective database. The study protocol was approved by the hospital ethics committee and a written informed consent was obtained from each patient before entering the study. Patients with preexisting infection, chronic renal insufficiency requiring dialysis, and on corticosteroids treatment were not included. Since CRP is synthesized in the liver, which is also the major source of PCT, patients with significant liver dysfunction (AST and ALT ≥ twice the reference value, prothrombin time ≤0.6, or macroscopic finding of liver cirrhosis or multiple metastases at laparotomy) were not included.

The following patient- and surgery-related data were recorded: demographic characteristics, comorbidities, American Society of Anesthesiologists (ASA) status, surgical diagnosis and procedure, duration of operation, and intraoperative and postoperative transfusion. Laboratory data determined preoperatively and on POD 1, 2, 3, and 5 included WBC count, hemoglobin, CRP, and PCT. All patients received antibiotic prophylaxis with gentamycin and metronidazol at standard doses for 48 hours. Thromboprophylaxis with low molecular weight heparin nadroparine 0.4-0.6 mL s.c. was started the evening before the surgery and continued postoperatively unless there was a reason for delaying thromboprophylaxis, ie, excessive bleeding or abnormal tests of hemostasis. Postoperatively patients were admitted to the surgical ICU for a minimum of 24 hours. In the ICU and on the ward, patients had regular clinical assessments and body temperature measurements. Additional laboratory and radiological investigations or endoscopic procedures were performed as indicated clinically. Patients were followed for the development of postoperative complications for a minimum of 15 days and the duration of hospital stay was recorded. They were seen in the outpatient clinic 7-10 days after discharge, when potentially missed complications due to an early discharge could be discovered.

### Definition of complications

Postoperative infectious complications were classified as surgical site infections (SSI) and remote infections. SSIs included wound infection, intraabdominal collection/abscess, and anastomotic leak (24). Wound infections were diagnosed in the presence of phlegmonous inflammation or purulent drainage from the wound. Intraabdominal collection/abscess and anastomotic leak were confirmed by contrast enhanced multislice CT scan, endoscopy, or during surgical exploration. Remote infections were pneumonia, urinary tract infection, and central venous line infection. Pneumonia was diagnosed by pulmonary infiltration on chest x-ray, accompanied by clinical symptoms and signs of lower respiratory tract infection. Urinary tract infection was diagnosed by a positive urine culture (>10^5^ colony forming units/mL urine). Diagnosis of the central venous line infection required positive blood cultures and cultures from the catheter tip.

### Biochemical analysis

The WBC count (reference range 3.4-9.7 × 10^9^/L) was analyzed using an automated hematological blood analyzer (Sysmex XE 2100, Sysmex, Kobe, Japan). The CRP concentration (reference range 0-10 mg/L) was determined by latex immunoturbidimetric method (Olympus AU 400, Olympus, Tokyo, Japan). The PCT concentration was measured by electrochemiluminescence immunoassay using Elecsys Brahms PCT kit (Cobas E 411, Roche, Germany). The detection limit of the assay was 0.02 µg/L and the upper limit of normal for hospitalized patients was 0.5 µg/L.

### Study size

Based on perioperative CRP concentrations in relation to the development of infectious complications after colorectal surgery (10,12,25), we assumed the diagnostic accuracy of 75% for early postoperative CRP concentrations. If we consider diagnostic accuracy of 85% as clinically relevant for a better biochemical marker compared to CRP, with the level of significance of 5% and the study power of 80%, each group should include 59 patients.

### Statistics

Statistical analysis was performed using STATISTICA data analysis software system (STATSOFT Inc, Tulsa, OK), version 9 and MedCalc Software version 12.1.3. (Mariakerke, Belgium). χ^2^ test was used to compare categorical variables. Numerical variables were tested for the normality of distribution using Kolmogorov-Smirnov test and were presented as mean ± standard deviation or median (interquartile range) as appropriate. *t* test was used to compare normally distributed variables, while nonparametric Mann-Whitney U-test was used to compare WBC count, CRP, and PCT values between the groups because PCT data were not normally distributed. Diagnostic accuracy of CRP and PCT concentrations for prediction of postoperative complications on each postoperative day was assessed by receiver operating characteristics (ROC) curve analysis (26). The best cut-off value was defined as the test result with the highest sensitivity and specificity and that lied closest to the left upper corner of the curve. The area under the curve presented a direct measure of the diagnostic accuracy of the test. The areas under the ROC curves were compared using the Z statistic (two-tailed test). A *P* value <0.05 was considered significant.

## Results

The study included 80 patients (51 male and 29 female) who underwent elective resection of the colon or rectum. One patient with macroscopic finding of liver cirrhosis at laparotomy postoperatively experienced worsening of liver function and was excluded from the study. Of the analyzed 79 patients, 50 had an uncomplicated postoperative course and 29 (36.7%) developed infectious complications. There were no cases of cardiovascular, thromboembolic, or other non-infectious complications. Cancer was by far the most common indication for surgery (92.4% of all patients), which was performed by an open method except for 3 laparoscopic procedures in the group without complications and one in the group with complications. In the group with complications significantly more patients underwent resections of the rectum (72.4% vs 32.0%, *P* = 0.001) and more patients received perioperative blood transfusion (75.9% vs 50.0%, *P* = 0.036) with greater volumes of blood transfused per patient. Twenty nine patients developed infectious complications after a median time of 7 days (range 5-14 days). Complications were wound infection (n = 10), wound dehiscence (n = 2), intraabdominal or pelvic collection (n = 7), anastomotic leak (n = 5), urinary tract infection (n = 2), pneumonia (n = 1), and central venous line infection (n = 2). One patient who underwent relaparotomy for a wound dehiscence and intraabdominal abscess died on the POD 10 from septic shock. The duration of hospitalization was significantly longer in patients with complications (22.3 ± 9.6 days vs 12.9 ± 2.3 days, *P* < 0.001), which implies higher costs of medical treatment of these patients (Table 1).

**Table 1:** Patient characteristics, surgery related data, and the duration of hospital stay. Values are mean ± standard deviation or number (percentage)

	No complications (n = 50)	Complications (n = 29)	*P*
Age (years)	65.7 ± 11.5	63.6 ± 11.3	0.434^†^
Sex (male:female)	25:25	25:4	0.002^‡^
Weight (kg)	75.0 ± 11.7	79.8 ± 9.4	0.063^†^
ASA status:			
2	39 (78.0)	20 (69.0)	0.536^‡^
3	11 (22.0)	9 (31.0)	
Preoperative hemoglobin (g/L)	131.5 ± 17.0	133.8 ± 17.8	0.571^†^
Cancer	46 (92.0)	27 (93.1)	0.793^‡^
Metastases/locally advanced disease	8 (16.0)	6 (20.7)	0.825^‡^
Surgical procedure:			
resection of the colon (ascending, descending, transverse, sigmoid)	27 (54.0)	6 (20.6)	0.005^‡^
resection of the rectum with colorectal anastomosis			
	13 (26.0)	9 (31.0)	0.828^‡^
abdominoperineal resection	3 (6.0)	8 (27.6)	0.019^‡^
Hartmann procedure	1 (2.0)	2 (6.9)	0.629^‡^
other procedures	6 (12.0)	4 (13.8)	0.728^‡^
rectal surgery (total)	16 (32.0)	21 (72.4)	0.001^‡^
Duration of surgery (min)	139.1 ± 57.3	159.0 ± 50.7	0.126^†^
Transfusion of PRBC:^§^			
number of patients	25 (50.0)	22 (75.9)	0.036^‡^
transfused volume (mL)/patient	525.2 ± 282.8	826.4 ± 666.1	0.045^†^
Hospital stay (days)	12.9 ± 2.3	22.3 ± 9.6	<0.001^†^

*Abbreviations: ASA – American Society of Anesthesiologists; PRBC – packed red blood cells.

^†^*t* test.

^‡^χ^2^ test.

^§^Intraoperative and postoperative days 0-2.

### Inflammatory markers

On POD 1, WBC count increased in patients with and without complications and then gradually declined. There was no significant difference in WBC count between the two groups at any measured time interval (Table 2).

**Table 2:** White blood cell counts in patients with and without complications. Values are median and interquartile range.

White blood cell count × 10^9^/L	No complications	Complications	*P^†^*
Preoperative	6.9 (5.8-8.2)	6.5 (5.6-8.1)	0.653
POD 1*	11.5 (10.4-12.8)	11.8 (10.0-13.7)	0.680
POD 2	9.7 (8.2-11.6)	9.3 (8.0-10.4)	0.173
POD 3	8.1 (6.5-9.7)	8.4 (7.4-9.2)	0.535
POD 5	7.1 (5.8-9.4)	7.6 (5.9-10.5)	0.317

*Abbreviations: POD – postoperative day.

†Mann-Whitney test.

**Figure 1:**
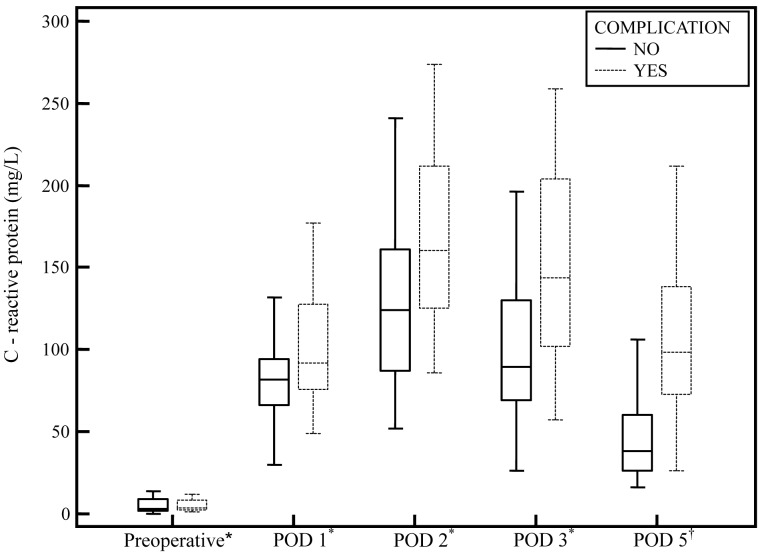
C-reactive protein (CRP) concentrations the day before surgery and on postoperative days (POD) 1-5 in patients with and without complications after colorectal surgery. The box represents 25th-75th percentiles and line within the box is the median value. *P* = 0.026 on POD 1, *P* = 0.002 on POD 2, *P* < 0.001 on PODs 3 and 5 (Mann-Whitney test). Asterisk indicates the group without complications n = 50; group with complications n = 29. Dagger indicates the group without complications n = 42; group with complications n = 27.

Preoperative CRP concentrations were not significantly different between patients with and without complications. Postoperatively, there was a sharp rise in CRP concentrations, reaching a peak value of 122 mg/L (interquartile range 87-161) in patients without complications and a peak value of 173 mg/L (interquartile range 143-230) in patients with complications (*P* < 0.001). CRP concentrations gradually declined in both groups from POD 3 to POD 5, more markedly in the group without complications (Figure 1). Importantly, CRP concentrations were significantly higher in patients with complications in all postoperative time intervals (*P* = 0.026 on POD 1; *P* = 0.002 on POD 2, *P* < 0.001 on POD 3, 5).

**Figure 2:**
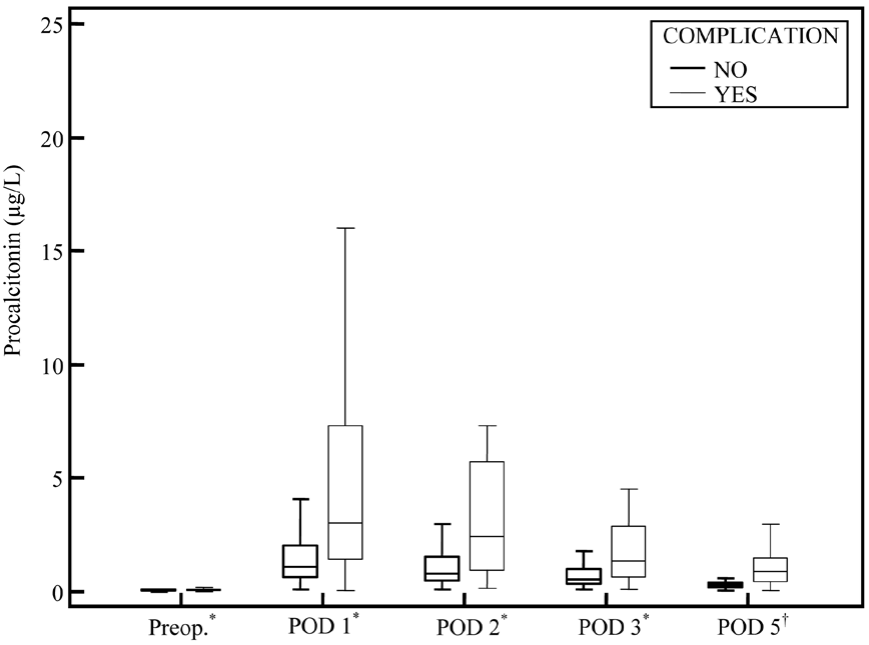
Perioperative procalcitonin (PCT) concentrations in patients with and without complications after colorectal surgery. The box represents 25th-75th percentiles and line within the box is the median value. *P* = 0.003 on postoperative day (POD) 1, *P* ≤ 0.001 on PODs 2-5 (Mann-Whitney test). Asterisk indicates the group without complications n = 49; group with complications n = 29. Dagger indicates the group without complications n = 37; group with complications n = 21.

Preoperative PCT concentrations were within the reference range in both groups of patients. Postoperatively, PCT increased significantly in both patients with and without complications, reaching peak values earlier than CRP (Figure 2). Maximum PCT concentration in patients with complications (2.3 µg/L, interquartile range 1.46-7.3) was significantly higher than in patients without complications (0.99 µg/L, interquartile range 0.62-1.71, *P* < 0.001). Similarly to CRP, PCT concentrations gradually declined from POD 2 to POD 5, being higher in patients with complications in all postoperative time intervals (*P* = 0.003 on POD 1, *P* ≤ 0.001 on PODs 2-5). Only in patients without complications PCT was within the reference values on POD 5.

**Figure 3:**
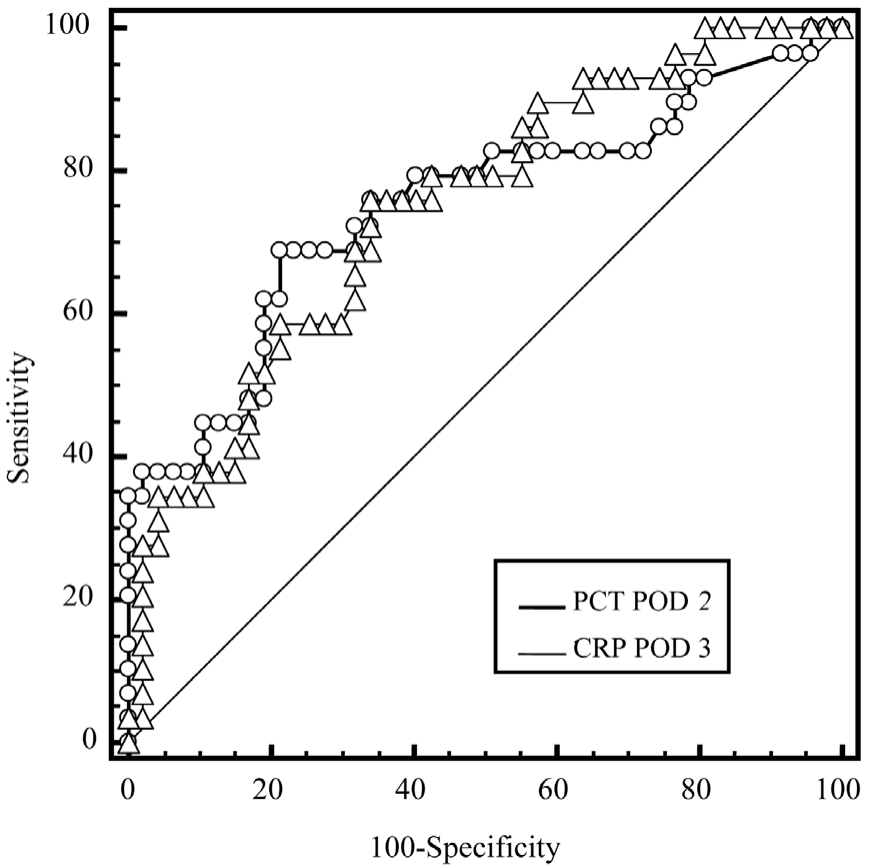
Diagnostic accuracy of early postoperative (postoperative day [POD] 1-3) C-reactive protein (CRP) and procalcitonin (PCT) concentrations with regard to infectious complications expressed by the receiver operating characteristic (ROC) curves. Comparison of ROC curves with the largest area under the curve for each biomarker on PODs 1-3 shows similar diagnostic accuracy of CRP on POD 3 and PCT on POD 2 with the area under the curve of 0.746 and 0.750, respectively.

To assess the predictive value of the serial postoperative CRP and PCT measurements for complications after colorectal surgery, we used ROC curve analysis of each inflammatory marker on each postoperative day (POD 1-3,5). Diagnostic accuracy of CRP for the prediction of postoperative complications was best on POD 3 (area under the curve of 0.746 [95% confidence interval 0.633-0.839]; *P* < 0.001) and for PCT on POD 2 (area under the curve of 0.750 [95% confidence interval 0.637-0.842]; *P* < 0.001) without significant difference between the areas under the two ROC curves (*P* = 0.963) (Figure 3). CRP threshold value of 99 mg/L on POD 3 provided a sensitivity of 75.9% and a specificity of 68.0%, while PCT threshold value of 1.34 µg/L on POD 2 provided a sensitivity of 69.0% and a specificity of 78.7%. Diagnostic accuracy of CRP and PCT was better on POD 5 than on POD 1-3, with the area under the curve of 0.851 for both CRP and PCT and the best cut-off values of 48 mg/L for CRP and 0.42 µg/L for PCT.

## Discussion

This study showed an increase in CRP and PCT in the early postoperative period after colorectal surgery, with a significant difference between patients with and without complications. ROC curve analysis showed that early postoperative CRP and PCT concentrations on POD 3 and POD 2 respectively, had similar predictive values for the development of postoperative infectious complications.

Mustard et al (27) found that serial postoperative CRP measurements predicted postoperative septic complications prior to clinical diagnosis in patients undergoing intraabdominal or thoracic procedures. This has been confirmed in more recent studies among patients undergoing colorectal and esophagogastric resections (10-15,25). Despite different patient populations and designs, they all have shown that high CRP concentrations that were persistently increased after POD 3 had a good diagnostic accuracy for prediction of infectious complications, especially anastomotic leaks. The median POD of clinical diagnosis of complications in these studies was 6-9. CRP measurements could therefore decrease the time to diagnosis and treatment of complications.

Our study showed that postoperative CRP concentrations in patients who developed complications were significantly higher than in patients with normal recovery. This is consistent with previous studies that examined all the infectious complications after elective resections of the colon or rectum (10,12,25). They reported good predictive values of CRP for postoperative infectious complications with cut-off values of 140 mg/L and 145 mg/L on PODs 3 and 4, respectively, in two studies and 125 mg/L on POD 4 in one study. This was higher than the CRP threshold value of 99 mg/L found in our study, but CRP concentrations of 80-100 mg/L have been used to differentiate sepsis from non-infectious SIRS in medical and surgical ICU patients (5,28). Lower diagnostic accuracy of CRP on POD 3 (area under the ROC curve of 0.746) with lower cut-off value than obtained in other studies (10,25) can be explained by rather low anastomotic leak rate in our patients. Intraabdominal infections, especially anastomotic leaks, have been associated with higher postoperative CRP levels than extraabdominal infections and better CRP predictive values (11,13,14). ROC curve analysis showed that diagnostic accuracy of CRP for intraabdominal infections and other septic complications was as high on POD 3 as it was on POD 4 (10,13,25). Taking this into consideration, together with faster kinetics of PCT, which could be associated with even earlier predictive value of PCT for postoperative infectious complications, we decided to determine CRP and PCT concentrations on PODs 1-3 and 5.

PCT is also induced in response to surgical trauma with the greatest increase observed after major gastrointestinal surgery (8,29). This has been explained by transient bacterial contamination or by liberation and translocation of bacterial endotoxins during malperfusion of the gut. In patients undergoing elective colorectal and aortic surgery, PCT was found to be a better marker of postoperative infectious complications than CRP (18), but this study included only 7 patients with septic complications after colon surgery. More recently, PCT has been found to perform better than CRP in diagnosing postoperative infections after orthopedic, cardiac, and thoracic surgery (21-23). However, these patient groups were significantly different from patients undergoing colorectal surgery.

In our study, postoperative PCT concentrations in patients without complications were similar to those previously reported in patients undergoing major abdominal surgery (7,8,29). Patients with complications had significantly higher postoperative PCT at all time points. Diagnostic accuracy of early postoperative PCT concentration for prediction of septic complications was similar to that reported by Mokart et al (30) on POD 1 in patients undergoing major gastrointestinal or gynecological surgery for cancer. In our study, the best PCT cut-off value on POD 2 was 1.34 µg/L and in the study by Mokart et al it was 1.1 µg/L on POD 1. Of note is a significant dispersion of values of postoperative PCT in patients with complications in both studies, which could decrease the value of PCT as an infection monitoring tool in the postoperative period and justifies further investigations. The reported diagnostic accuracy of PCT for infectious complications after cardiac and thoracic surgery has been better than after gastrointestinal surgery (22,23). However, these studies included a relatively small number of patients with complications (16 and 25, respectively) and used maximum and mean PCT and CRP concentrations in the first 5-7 postoperative days to calculate the diagnostic accuracy by ROC curve analysis. Using serial inflammatory marker determinations might improve predictive value of the marker for detection of postoperative infections.

In our study, the best diagnostic accuracy of postoperative CRP and PCT (area under the ROC curve of 0.851 for both) was obtained on POD 5. Because the median day of clinical diagnosis of postoperative infections was 7 (range 5-14 days), we considered POD 5 as rather late time for “early” prediction of infectious complications. Also, the optimum cut-off values of CRP and PCT on POD 5 were lower than those commonly used for differentiation of infectious and non-infectious SIRS, which reduces the clinical value of the test.

Elevated WBC count is a nonspecific inflammatory marker and one of the SIRS criteria. Therefore, it is not surprising that WBC count had a poor diagnostic performance for infection in ICU patients and postoperatively (10-12,14,21,23). Some authors report a late increase in WBC counts in patients with infectious complications following colorectal surgery, correlating with the clinical diagnosis of complications (10). We could have missed the late increase in WBC count in patients with complications, since we assessed inflammatory markers only on PODs 1-5, aiming at an early detection of postoperative complications.

Our study with prospective data collection and patient evaluation provides important information on diagnostic performance of periopertive PCT compared to CRP for the development of infectious complications after colorectal surgery. The main limitation of the study is a relatively small number of patients with complications. Although our study does not have statistically sufficient power, a small difference of 0.4% between areas under the ROC curves for CRP on POD 3 and PCT on POD 2 with significance level *P* = 0.963 adds to the validity of the data.

According to our results, postoperative PCT determinations have similar diagnostic accuracy to CRP determinations. The earlier postoperative peak of PCT does not seem to offer the advantage for diagnostic purposes. Based on the study results and taking into account the higher cost of PCT test, we cannot recommend routine perioperative PCT measurements for the prediction of infectious complications following colorectal surgery.
